# Neonatal Immune System Ontogeny: The Role of Maternal Microbiota and Associated Factors. How Might the Non-Human Primate Model Enlighten the Path?

**DOI:** 10.3390/vaccines9060584

**Published:** 2021-06-01

**Authors:** Natalia Nunez, Louis Réot, Elisabeth Menu

**Affiliations:** 1 CEA, Université Paris-Sud, Inserm, U1184 “Immunology of Viral Infections and Autoimmune Diseases” (IMVA-HB), IDMIT Department, IBFJ, 92265 Fontenay-aux-Roses, France; nnunez@lifeandsoft.com (N.N.); louis.reot@cea.fr (L.R.); 2 MISTIC Group, Department of Virology, Institut Pasteur, 75015 Paris, France

**Keywords:** microbiota, immune system maturation, colonization, non-human primate, pregnancy, birth, breastfeeding, weaning, vaccination, probiotics

## Abstract

Interactions between the immune system and the microbiome play a crucial role on the human health. These interactions start in the prenatal period and are critical for the maturation of the immune system in newborns and infants. Several factors influence the composition of the infant’s microbiota and subsequently the development of the immune system. They include maternal infection, antibiotic treatment, environmental exposure, mode of delivery, breastfeeding, and food introduction. In this review, we focus on the ontogeny of the immune system and its association to microbial colonization from conception to food diversification. In this context, we give an overview of the mother–fetus interactions during pregnancy, the impact of the time of birth and the mode of delivery, the neonate gastrointestinal colonization and the role of breastfeeding, weaning, and food diversification. We further review the impact of the vaccination on the infant’s microbiota and the reciprocal case. Finally, we discuss several potential therapeutic interventions that might help to improve the newborn and infant’s health and their responses to vaccination. Throughout the review, we underline the main scientific questions that are left to be answered and how the non-human primate model could help enlighten the path.

## 1. Introduction

**Figure 1:**
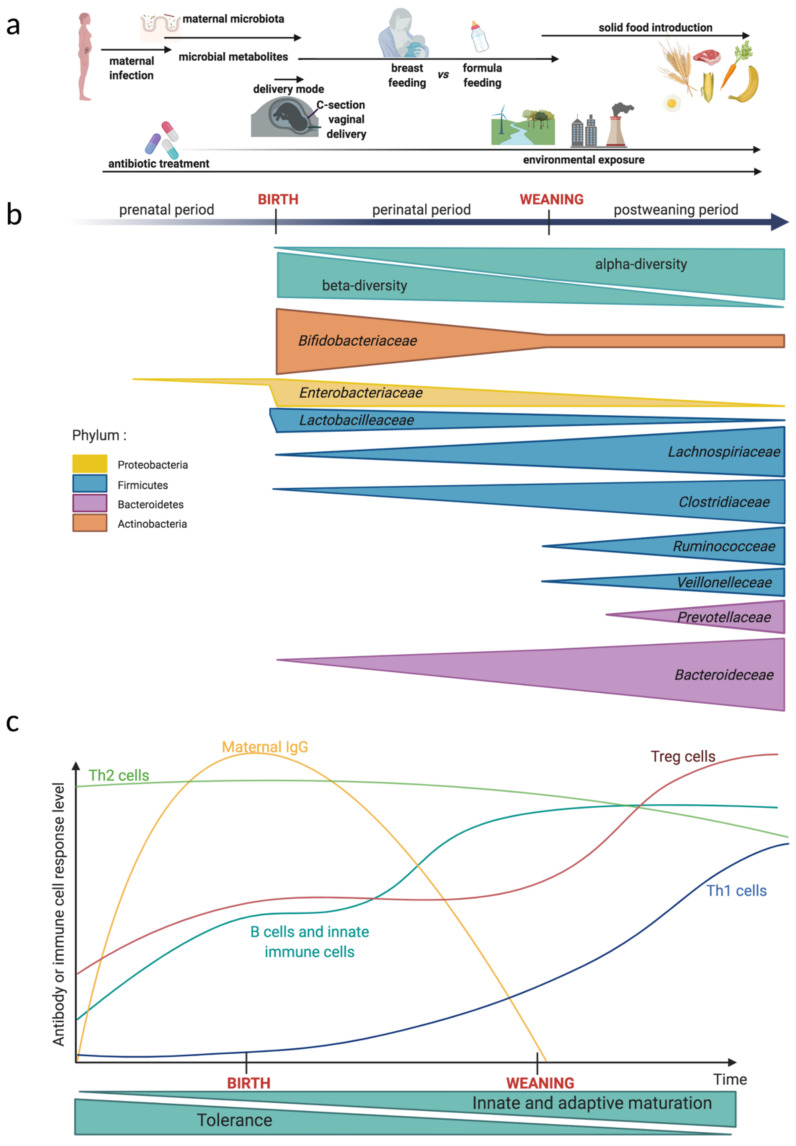
Kinetics of the local environment impact, the gut microbiota evolution, and the immune system maturation from fetal development to the neonatal period. (**a**) Factors affecting the evolution of the microbiota and the immune system. During the prenatal period and even after birth, maternal infection and antibiotic treatment disturbs fetal development. After birth, the gut microbiota and the development of the immune system are both influenced by environmental exposure. The mode of delivery and feeding impacts the colonization of the neonatal intestinal mucosa. Around weaning, the timing of solid food introduction and the composition of the diet determines the gut microbiota dynamics and the immune system maturation. (**b**) Evolution during the prenatal period and early childhood of the main bacteria families in the gut microbiota. The size of each triangle reflects the relative abundance of each bacteria family or the bacterial richness. Fetal colonization is still a matter of debate. Neonate gut presents a reduced microbial diversity at an individual level (α-diversity) but a high variation of microbial communities when compared between individuals (β-diversity). After weaning, the α-diversity strongly increases and the gut microbiota becomes more resilient. In this figure, bacteria belonging to the same phylum are grouped in the same color. (**c**) Immune system development kinetics in the prenatal period and early childhood. Innate and adaptive immune cells develop throughout the fetal period and early in life. Before and around birth, the fetal immune system is dominated by a pro-tolerogenic response and a Th2 cell polarization. Maternal IgG are transferred to the fetus before birth and confer a protective immunity to the neonate. The neonate B cells and innate immune cells are phenotypically similar to the adult before weaning. After weaning, the proportion of regulatory T cells (Tregs) increases and the infant presents a balanced lymphocyte Th1/Th2-polarization. Created by BioRender.com, accessed on 20 April 2021.

Mammal commensal microbes colonize most of their host’s surfaces including the skin and the mucosa. Neonatal microbial colonization depends on several factors that include, among others, the delivery mode, the environment, feeding, weaning timing, and antibiotic use (Figure 1a). The exposure of the newborn to microbial antigens facilitates the development and maturation of the immune system. In fact, the ontogenesis of the immune system begins as early as three weeks after conception and this process continues after birth and into childhood [1].

The mother’s microbiotas (vaginal, perineal, and intestinal) are among the initial factors shaping newborn colonization. Surprisingly, the impact of a mother’s dysbiotic microbiota on the child’s health outcome is still matter of study. The disruption of the microbiota colonization during the neonatal period and in early childhood has been linked to several chronic pathologies, which include obesity, asthma, and autoimmune diseases [2]. It is thus of great importance to dissect the association in the mother–newborn interphase. The hygiene hypothesis stipulates an association between low exposure to microorganisms in childhood and a high risk of autoimmune diseases and atopy [3].

**Figure 2:**
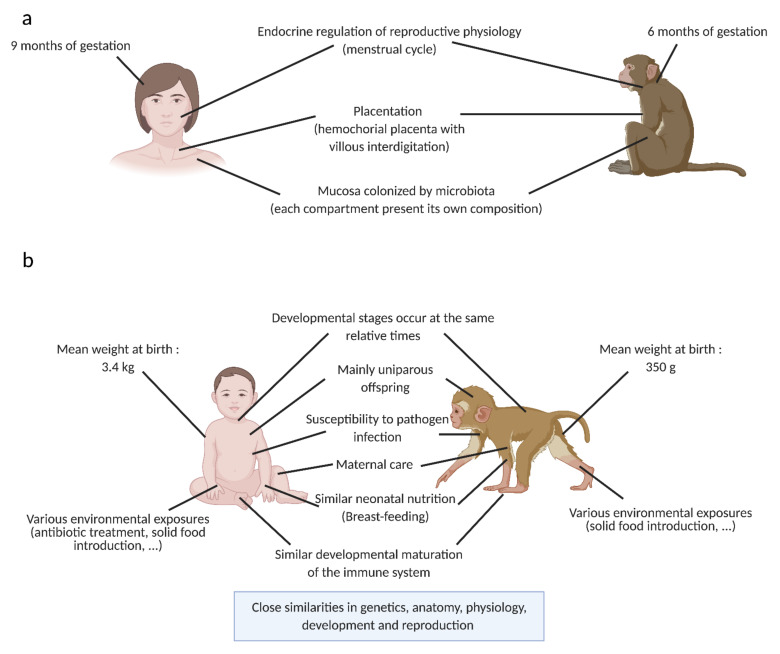
Comparison between the adult (**a**) or the infant (**b**) in human and in the non-human primate model: main characteristics relevant for the microbiota and the immune system study in neonates. Similar characteristics between models are shown in the middle of the figure. Specific characteristics for each model are shown in the lateral sides of the figure. Created by BioRender.com, accessed on 20 April 2021.

Animal models—in particular the non-human primate (NHP) model—are useful for exploring novel hypotheses and for answering fundamental questions that are difficult or impossible to explore in humans. The NHP model has several advantages as a research model, such as its proximity to human genetics, physiology, and immune system as well as their susceptibility to many human infectious diseases having similar pathogenesis [4,5]. These similarities renders it a solid model for fundamental research studies including immune development in early life. The NHP model has contributed to a better understanding in reproductive and developmental science [6]. It is also a model of choice for the development of novel vaccines and therapies for the prevention and treatment of human diseases. We have summarized the main similarities and differences between humans and NHPs that are relevant in the mother–infant interphase in Figure 2.

We will focus on the ontogeny of immune maturation and its association to microbial colonization in newborns from conception to the food diversification. We underline the questions that are left to be answered and how the NHP model could help enlighten the path to answering these questions.

## 2. Pregnancy: Mother—Fetus Interactions

The vaginal microbiota in reproductive-aged women is classified in five community state types (CST) [7], where CST I, II, III and V are dominated by *Lactobacillus crispatus*, *L. gasseri*, *L. iners*, and *L. jensenii*, respectively. CST IV does not have a dominant species and is characterized by having a very low to absent lactobacilli and high anaerobic bacteria abundance. CST IV is often associated with bacterial vaginosis and inflammation. In the NHP model and, in particular, in macaques the vaginal microbiota is dominated by diverse anaerobes and has a low lactobacilli abundance [8].

During pregnancy, the vaginal microbiota of healthy women changes. It has been demonstrated by Romero et al. that lactobacilli (*L. crispatus*, *L. jensenii*, *L. gasseri*, and *L. vaginalis*) abundances increase while anaerobes such as *Eggerthella*, *Dialister*, *Gardnerella*, and *Atopobium* abundances decrease as pregnancy progresses [9,10]. Pregnant women have less incidences of anaerobic bacteria that are associated with bacterial vaginosis and their vaginal microbiota possesses higher stability [9,10,11]. Interestingly, in Romero’s studies, the relative abundance of *L. iners* was neither significantly different between pregnant and non-pregnant women nor for women who delivered at term vs. women who delivered preterm. On the contrary, Aagaard et al., states that there is indeed an enrichment in lactobacilli (*L. iners*, *L. crispatus*, *L. jensenii* and *L. johnsonii*) during pregnancy [11]. We can thus believe that lactobacilli plays a role in vaginal microbiota homeostasis during pregnancy. However, the presence and beneficial role of *L. iners* in vaginal health remains a matter of debate. *L. iners* is in fact widely associated with an inflammatory, dysbiotic, and prone to infection vaginal environment [12,13]. Its only presence in pregnant women microbiota suggests that there might not be natural protection in pregnant women to infection or inflammation.

Antibiotic use is quite extensive during pregnancy [14,15], with reported rates of 42% in France, 40% in the US, 33% in the United Kingdom, and 24% in Finland [14,16,17]. Among antibiotics, β-lactams are the most widely used and penicillin is the most frequently used. Antibiotic use during pregnancy or during partum impacts both the mother’s microbiota on the birth canal and the vaginally delivered infant colonization [18,19]. For instance, the use of ampicillin for group B *Streptococcus* (GBS) treatment results in an increase in Proteobacteria and a decrease in Actinobacteria and Bacteroidetes in the gastrointestinal microbiota of vaginally born infants. These alterations decrease diversity in the gastrointestinal tract and persists up to twelve months after birth [18,20,21]. In addition, disruptions can increase the risk of neonate morbidity and negatively impact microbial colonization [19].

Maternal stress is another factor that impacts infant colonization. Gestational stress has a negative impact in fetus growth, weight, development, immunity, and cognitive function in infant rhesus primates (*Macaca mulatta*) [22,23]. In rhesus macaques, chronic stress also impairs prenatal transfer of antibodies to the fetus [24]. In addition, gestational stress alters colonization in the infant primate that possesses a reduced abundance of *Lactobacillus* and *Bifidobacterium* compared to infant primates from undisturbed mothers [25]. The vertical transfer of *Bifidobacterium*, in particular *B. bifidum*, has also been observed in humans [26]. The reduction in lactobacilli and bifidobacteria have both been linked to diarrhea, food allergy, and chronic inflammatory bowel diseases [27,28]. Consistent with this observation, infant macaques born after disturbed gestations have a tendency for *Shigella flexneri* colonization.

Maternal environment and microbiota play a role in immune maturation. Numerous epidemiological studies show that prenatal maternal exposure to a rural environment is associated to a rapid maturation of Th17 lymphocytes in children and to a decreased risk of developing allergies or asthma [29]. The mother’s vaginal microbiota plays an equal role in the fetus immune maturation where women with vaginal dysbiosis exhibit an increased concentration of interleukin 12 (IL-12) and a decreased concentration of CD45RO CD4+ T cells in the umbilical cord blood [30]. Indeed, endogenous lactobacilli present in the mother’s vagina may impact CD4+ T cell maturation and lead to the generation of memory T cells. Nevertheless, the mechanisms by which the fetal immune system recognizes and responds to the mother’s microbiota or to their metabolites are still unclear. The recognition process could be by leakage of bacterial antigens through the placenta. Exchanges of molecules across the placenta are well documented [31], particularly for selective transmission of maternal Immunoglobulin G (IgG) mediated by the neonatal Fragment crystallizable receptor [32]. Antigens also reach the amniotic fluid with a very low efficacy through passive diffusion or paracellular transport across the fetal membranes [33], where fetal swallowing activity contributes to antigen exposure in the fetus’ gastrointestinal tract mucosa.

During the late fetal period, when antigen presenting cells have reached mucosal tissues, antigens encountered can induce tolerance. This tolerance prevents an inflammatory response in the fetus to non-self-antigens, such as those from the mother or derived from food [34,35]. An important example of the importance of this exposure to non-self-antigens during pregnancy is provided by a study from Gomez de Aguero et al. showing that transient *E. coli* colonization of the gut of germ-free (GF) pregnant mice affects the offspring’s immune system during gestation, with an increase in intestinal innate immune cells and pattern of gene expression in their gut compared to control mice [36]. Interestingly, the authors do not detect any live bacteria in the placenta or in the neonate, which suggests that the mechanism for this immune cell maturation likely involves bacterial metabolites, namely in that study, ligands for aryl hydrocarbon receptor. Other bacterial metabolites might be equally involved in gestational microbial shaping of immunity [37]. Unfortunately, bacterial metabolites are poorly studied in neonates and even less in the context of shaping the immune system during pregnancy.

Besides the role of the mother’s intestinal and vaginal microbiota in neonate colonization, researchers questioned whether the upper female reproductive tract, the extraembryonic, and embryonic tissues were colonized by microbes. Potential microbial in utero colonization have to be clearly differentiated from the bacteria that are responsible for microbial invasion of the amniotic cavity (MIAC). Pathogens that are at the origin of MIAC are *Ureaplasma urealityticum*, *Mycoplasma hominis*, and *Fusobacterium* spp. [38]. This microbial presence, however, is considered as an intrauterine infection that has severe outcomes that include preterm deliveries, fetal infection, and neonatal sepsis [39,40,41]. In the case of intrauterine infection, bacteria or inflammatory mediators can be swallowed by the fetus to trigger fetal immune response characterized by elevated IL-6 concentration in the umbilical cord [42]. Fetus exposed to inflammation also have monocyte, neutrophil, and lymphocyte activation in addition to a Th1-like response and a secretion of tumor necrosis factor α (TNF-α), interferon γ (IFN-γ), IL-1β, and prostaglandins that can be traced to the origin of premature membrane rupture as well [43].

A commensal or symbiotic flora, by definition, does not have a negative outcome for mothers or for the children. Several studies published later show that bacterial DNA is present in the uterus, umbilical cord, placenta, meconium, and the fetus [44,45,46,47,48]. It is Proteobacteria that is the most prevalent phylum in amniotic fluid and placenta samples with *Enterobacter* and *Escherichia*/*Shigella* being the predominant genera [44,45]. *Escherichia*/*Shigella* and lactic acid bacteria (*Leuconostoc*, *Enterococcus Lactococcus*, *Staphylococcus*, and *Streptococcus*) were also observed in meconium (the earliest stool sample of a newborn) but in lower abundance. Meconium microbiota cluster into two enterotypes. Those that are dominated by lactic acid or those dominated by enteric bacteria. These enterotypes are associated with respiratory problems in infants and further associated to a history of maternal eczema, respectively [46]. These results suggest that the neonate colonization has a uterine origin. However, most of these results rely mainly on DNA sequencing. Additionally, to avoid invasive procedures, samples are generally collected after delivery or C-section, which can put into question the sample’s sterility. The observed results might be the amplification of contaminant DNA (laboratory or hospital environment linked to the sampling procedure) or the result of bacteria DNA presence in the uterus. DNA presence does not necessarily mean that there are metabolically active living bacteria. A recent study on rhesus macaques including not only sequencing but also bacterial culture and qPCR showed that cultures from fetal and placental tissues were very rare and inconsistent with an in utero colonization [49]. In fact, only *Cutibacterium acnes* was isolated from bacterial culture and the 16s rRNA gene sequence was detected in the umbilical cord and fetal tissues. However, *C. acnes* DNA was also detected in the technical controls indicating that its presence might be due to contamination from the manipulator.

Currently, it is still controversial as to whether human colonization starts in utero and fetal microbial colonization remains an uncertain topic of discussion. What is equally important to highlight is that microbial antigens (including DNA) and food derived and maternally derived molecules can be found in the amniotic fluid [50,51,52]. Those antigens could be directly transferred into the amniotic fluid through passage across the fetal membranes [53]. All of these observations suggest that maternal prenatal factors can have a critical impact in the development of child health and disease.

The fetus has innate and adaptive immune cells that are present early during pregnancy. The development of the human immune system is in fact a continuous process that begins in utero. As pregnancy develops, the antigen presenting cells are formed: dendritic cells and monocytes are the first innate immune cells to appear around the fourth gestational week (GW). The thymus develops and matures between the GW10 and GW14, followed by the spleen and lymph nodes between GW14 and GW20. B and T cells largely naive in phenotype are detected around GW10. T and B zones in the spleen are formed in the middle of the second trimester (around GW20). Myeloid progenitors are abundant in fetal blood but their differentiation occurs during the late stage of pregnancy: mature neutrophils are the last cells to appear in fetal blood and their concentration increases just before birth, which is concomitant with increase in G-CSF levels [54].

During development, the fetus is exposed to several antigens that include both maternal and exogenous antigens. Prenatal exposition occurs via the placental barrier or via the amniotic fluid that is ingested by the fetus [34,35]. Importantly, transplacental transport of maternal Ig-antigen complexes can result in the priming of fetal immune cells [50]. The fetus is in fact able to generate an immune response. It has indeed been shown that it can respond to exogenous antigens, such as infectious agents or vaccines [55,56], but its response generates a low level of proinflammatory cytokines compared to neonates or adults where the number of interleukin 6 (IL-6) and TNF-α positive monocytes were significantly diminished. Fetal phagocytes display a significantly diminished phagocytic capacity and the expression of several adhesion molecules involved in adhesion, migration, and phagocytosis was simultaneously found to be decreased [57]. Conventional dendritic cells (cDCs) and plasmacytoid dendritic cells (pDC) are impaired in their IL-12 and IFN-α secretion, respectively, and fetal mononuclear cells also possess a reduced interleukin 2 (IL-2) production that is essential to T cell function [58]. T cell progenitors may express CD34 receptor and will migrate to the thymus to differentiate to mature CD4 or CD8 cells. T helper cells can be divided in several subsets including effector Th1 and Th2 CD4+ cells, which is defined by their cytokine profiles and regulatory T cells (Tregs). On one hand, Th1 cells are pro-inflammatory and secrete IFN-γ, IL-2 and TNF-α. On the other hand, Th2 cells produce cytokines such as IL-4, IL-5, IL-13, and IL-10 that are mainly anti-inflammatory and pro-tolerogenic. Tregs are present in very high numbers in human cord blood and have an immunosuppressive function. The low level of proinflammatory cytokines produced by the fetus indicates that fetal immune responses are mainly immune-suppressing rather than immune-activating. In summary, fetal lymphocytes exhibit a preferential differentiation towards pro-tolerogenic cells and a Th2 response for T cells, allowing the formation of a repertoire of self-antigen tolerated by the fetal immune system. Maternal cells also contribute to the formation of the antigen-specific tolerance repertoire since they cross the placental barrier [35]. This tolerance is essential for avoiding potentially damaging immune reactions from the fetus towards maternal antigens and promotes the positive progress of pregnancy. In contrast, at birth, the neonatal immune system will have to switch from a predominantly tolerogenic response to an anti-microbial response suitable for protecting the newborn from the new microbe-rich environment after birth [3].

Altogether, these ideas support the fact that the maternal environment is crucial to the proper development of the fetus, the exact mechanisms of the triptych interaction between the mother, her microbiota, and the fetus for the development of fetal immune maturation remain to be elucidated. In particular, the modalities of antigen exposure that determine immune system maturation remain an enigma. To give two examples, on one hand, fetal memory T cells are produced during uncomplicated pregnancies [54] and, on the other hand, neonates that have been exposed to maternal infection during pregnancy, but were not infected, developed an adaptative immune response to the pathogen [59]. In both cases we cannot explain the biological mechanisms for these phenomena.

**Figure 3:**
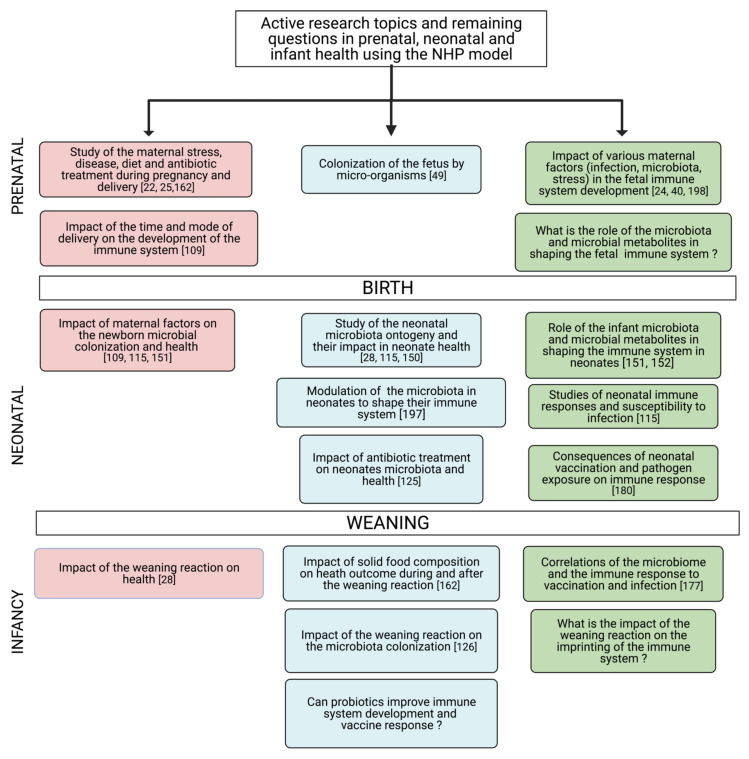
Summary of active research topics and questions to address the relationship between the microbiota and the immune system in the NHP model. Pink boxes represent the mother or parental-associated parameters, blue boxes represent microbiota studies, and green boxes represent immune system studies. The numbers represent the main references addressing the issue in the literature. Created by BioRender.com, accessed on 20 April 2021, [22,24,25,28,40,49,109,115,125,126,150,151,152,162,177,180,197,198].

Studies in NHP models would allow the investigation of many of these questions that are impossible to address in other models. For example, it would be possible to elucidate the possible role of various maternal factors (i.e., microbiome or infection) in the fetal immune system development. Indeed, in the NHP model, longitudinal follow-up of the maternal microbiome in various compartments and the observation of bacterial metabolite distribution during pregnancy or mucosal immunity in the developing fetus would be possible [60]. The NHP model would also offer an excellent preclinical model to test various strategies for treatments during pregnancy while simultaneously targeting an optimal fetal immune system development (Figure 3).

## 3. Birth

During birth, for uncomplicated pregnancies, the newborn goes from a relatively aseptic environment to an environment that is highly diverse with an abundance of microorganisms. Faced with this brutal increase in microbial exposure, the newborn has to be able to control microbial colonization to avoid invasion by pathogens. Factors that influence microbial colonization and immune response to this colonization at birth include the mode of delivery and gestational age (GA) at term (Figure 1a).

### 3.1. Preterm vs. Term Delivery

In humans, preterm birth is defined as babies born alive before 37 weeks of pregnancy. Prematurity is the leading cause of death in children under the age of 5 years. The rate of preterm birth ranges from 5–18% and this rate is increasing worldwide (WHO, 2018). Preterm birth occurs for multiple reasons, from chronic disease in the mother (i.e., diabetes or preeclampsia) to multiple pregnancies or infections. However, most preterm births are idiopathic. The rupture of membranes is the opening of the membranes (amniotic sac) before labor. If this rupture occurs before 37 weeks, it is considered a premature rupture. A premature membrane rupture is often due to inflammation and pathogen colonization of the maternal and fetal tissues (choriodecidual space, chorion, amnion, placenta, amniotic fluid, umbilical cord, and the fetus itself). Bacteria invasion may have multiple origins such as hematogenous spread from the mother, needle contamination during amniocentesis, or ascension from the vagina. Preterm labor with intact membranes is associated to a vaginal dysbiosis [61,62].

Several clinical studies that followed women experiencing spontaneous and not infectious preterm birth and women who delivered at term showed that spontaneous preterm birth is associated to an increased vaginal bacterial diversity [61,62,63,64]. Most of the commonly identified vaginal bacteria in preterm birth are *Mollicutes*, *Meagaspharea*, *Prevotella*, *Sneathia*, *Ureaplasma*, *Mycoplasma*, *Gardnerella*, *Streptococcus*, and *Bacteroides* [61,62,64,65]—all bacteria that are associated to a dysbiotic vaginal microbiota [7]. Interestingly, women that delivered at term are more likely to have an *L. crispatus* dominated microbiome [61,62,64].

At the time of birth, the neonate adaptive immune system is not fully mature and has to develop memory. Compared to adults, neonate immune cells harbors a different phenotype and different functions [66]. For example, neonate monocytes are poorly reactive to lipopolysaccharide (LPS) stimulation compared to adult monocytes [67]. The neonate’s lymphocytes have a differentiation bias toward Th2 response [68] (Figure 1c), which makes the neonates more susceptible to various infections, especially in preterm children [69,70]. The full-term neonates are perfectly capable of triggering both innate and adaptative immune responses [71], however, they trigger a response that is qualitatively and quantitatively different from adults [72]. In the case of preterm babies, the phenotype and function of immune cells are altered compared to full-term children [43]. It has been observed that preterm children overexpress genes involved in the negative regulation of IFN-γ production and T cell proliferation, as well as the genes associated with IL-10 secretion [73]. These immune signatures in preterm babies can result in higher susceptibility to disease, deficient viral detection, and clearance [74]. Furthermore, preterm children possess decreased levels of maternal IgG compared to full-term children and this reduction is linked to the severity of their prematurity. Indeed, there is an exponential relationship between total IgG and GA in preterm infants [75]. More generally, GA appears to be an important parameter that influences immune cell differentiation in the neonate and not only in preterm infants [76]. Indeed, even if several studies mentioned before show great differences in immune cell populations or cytokine profiles between preterm and full-term children at birth, those differences are lost as the child grows [73].

### 3.2. Mode of Delivery: C-Section vs. Vaginal Delivery

Both the place and mode of delivery are critical in shaping the infant microbiotas [77]. Cesarean section (C-section) is a surgical intervention intended to reduce health risks of mothers and their babies when vaginal birth is considered unsafe. However, in some countries, C-section is a choice and a number of women request to deliver by elective C-section without medical indications [78]. It is important to highlight that the effects of C-section on perinatal morbidity, pediatric outcomes, and disease are still unclear. Present research focuses mainly on the immediate effects of C-section relative to the infant and the mother’s health.

There is evidence that the gastrointestinal tract of infants born either by C-section or vaginally are different not only in their composition but also on the timing of their colonization. During the first week of life, bacteria in the stool of vaginally delivered babies ranged from 10^9^ to 10^10^/g of feces, whereas C-section born babies had lower bacterial counts (<10^8^ rRNA copies/g of feces) [79]. Vaginally delivered babies have an enrichment of *Lactobacillus* followed by a bloom of *Bacteroides* from week two after birth [80]. This was also observed in rhesus monkeys, where *Lactobacillus* was present during the first six months of life in naturally-born infant monkeys [25]. *Bifidobacterium* increases with a peak at eight weeks after birth but declined with the onset of weaning and the introduction of solid food [25]. Vaginally delivered newborns also harbor bacteria like *Enterococcus*, *Escherichia*, *Streptococcus*, and *Rothia* [77]. Altogether, these results indicate that vaginally born neonates are inoculated by a mix of Gram positive and Gram negative bacteria that are both facultative aerobic and anaerobic bacteria.

C-sections are often associated to antibiotic use, low baby-mother contact, an underlying medical condition, pregnant pathologies, and a delay in breastfeeding [81,82]. All of these conditions, in addition to the C-section itself, result in a decreased bacterial diversity and richness in the gut characterized by a lower *Lactobacillus*, *Bifidobacterium*, and *Bacteroidetes* abundance in infants [80,83,84,85,86,87]. C-section born babies also have an increase in *Clostridioides*, *Enterobacter*, *Klebsiella*, *Haemophilus*, *Staphylococcus*, *Streptococcus*, and *Veillonella* abundances [77,86,87], suggesting that skin and oral bacteria are the first colonizers in C-section born infants instead of bacteria from the mother’s vaginal microbiota.

The differences in neonate microbial colonization and, in particular, the reduced diversity may be one of the explanations for the higher incidence of immune diseases and high inflammation in children born by C-section and in premature children [46,71,85,88]. In fact, decreased abundance of *Bacteroidetes* and *Bifidobacterium* is associated with the development of allergic or auto immune diseases. Interestingly, a reduced bacterial diversity is also found in breast milk from women who delivered through C-section compared to those who delivered vaginally [89,90].

The importance of the neonate colonization and the long-term health consequences between C-section and vaginally born infants remain unclear. Numerous epidemiological studies made an association between disturbances of the microbiota in childhood, associated with antibiotic treatments during pregnancy, and the development of a pathology in adulthood [91]. In fact, we know that intestinal disruptions are associated with immune diseases and chronic infections such as inflammatory bowel diseases [92,93,94], obesity [25,86,95], and asthma [96,97] in adults. However, it is difficult to tell whereas C-section is fully detrimental for children and its full impact on children’s health remains unclear. A study in mice showed that the response of intestinal epithelial cells to LPS stimulation is stronger in vaginally born mice than in newborns by C-section [98]. In humans, studies on the relationship between C-section and the infant immune system have been inconsistent. Some studies suggest that there is no definite correlation between C-section born babies and the development of allergic rhinitis or atopic dermatitis [99,100,101], while others show that C-section is indeed associated with decreased pro-inflammatory cytokine response to Toll-like Receptor (TLR) 1 and 2 stimulation, a high bacterial colonization in the airways during late infancy, and a higher risk for infantile wheezing [102]. Furthermore, in children with increased genetic risk for type 1 diabetes, C-section is associated with transcriptional changes in blood cells and correlates with the initiation of islet autoimmunity, which precedes type 1 diabetes [103]. Other studies strongly indicate that there is also an association between C-section and childhood obesity [104]. In fact, C-section born babies might have a microbiota prone to harvest more dietary nutrients, therefore predisposing them to being overweight. In addition, the absence of breastfeeding predisposes babies to excess adiposity and an increased Body Mass Index [105,106]. In another study, birth by C-section was associated with an increased risk for infection-related hospitalization [107]. Although such associations exist, the mechanisms for the relationships between the mode of delivery, the immune system maturation, and the disease apparition later in life remains largely unknown. This might be because postnatal factors are not always available in human studies. However, we do know from animal studies if the mode of delivery can impact host immunity directly [108]. The NHP model could be useful to address the impact of the mode of delivery on the development of the immune system in longitudinal studies taking into account the temporal dynamics of vaginal microbiota and other confounding factors [109].

In order to overcome potential health problems that result from a disturbed colonization at birth, physicians are now aiming to restore the microbiota of C-section delivered babies [80]. In a pilot clinical study, C-section delivered babies were inoculated with healthy vaginal microbiota (absence of GBS, no signs of vaginosis and vaginal pH below 4.5) from their mothers [80]. The microbiomes (anal, oral, and epithelial) of these treated infants resembled those of vaginally delivered infants during the first week after birth. As long as the mother’s vaginal microbiome is characterized, these approaches might be a temporal solution to overcome the current unclear links between birth mode and future detrimental health outcomes in the child.

To summarize, colonization of mucosal surfaces at birth is mediated by different bacteria depending on the gestational age at birth and on the mode of delivery. The great diversity in those communities between individuals is directly translated by a great β-diversity at birth. Depending on the colonization, the different microbial populations induce various stimulations on immune cells. It is important to underline that the immune system is relatively naive at birth and does not respond strongly to antigen stimulations. This low reactivity provides a form of protection of the neonate against a strong immune reaction that could damage their good development [110,111,112,113]. Exposition to microorganisms at the beginning of life is important for permanently shaping the immune system [114] and for reducing the risk for developing immune-mediated pathologies later in life [91]. The NHP model allows the study of the acquisition of the microbiota and the associated maturation of the immune system in controlled settings, which enlightens the contribution of the numerous factors involved at the materno–neonatal interface [115].

## 4. Neonate Colonization and Role of the Breastfeeding

As shown in the previous chapters, the infant’s gastrointestinal microbiota colonization at birth is directly linked to the mother’s microbiota where the first colonizers of the baby are the bacteria from the mother. However, this first colonization is not stable in terms of time and many factors from the environment (nutrition, neonatal infection, antibiotic use, and local environment such as the presence of pets and pollution) impacts colonization and the early development of the immune system [116] (Figure 1a). Diet is known to be the main factor in shaping the gut microbiota in adults; it is also the main factor influencing the gut microbiota during the first six months of life [117]. As soon as lactation begins, novel microorganisms from the breast milk will be carried into the gut (Figure 1b). The importance of the intestinal microbiota (either the mother’s or the neonate’s) on the development of the immune system in newborns is perhaps best illustrated by GF or deliberately colonized gnotobiotic animals. It has been demonstrated that the microbiota improves digestion, stimulates the development of lymphoid structures, regulates innate and adaptive immune systems, provides colonization resistance against pathogens, and shapes the development of the nervous system and its behavior [118]. GF mice exhibit major developmental defects in primary and secondary lymphoid organs (Gut-associated Lymphoid Tissue (GALT), spleen, and thymus), in addition to abnormalities in the frequency, phenotype, and function of immune and intestinal cells [119,120,121]. To mention a few, GF intestines have reduced absorption of ingested materials with a reduced surface area of small intestines, altered enzyme expression and increased bilirubin in feces, reduced activation in intestinal macrophages and IgA secretion, low systemic Ig levels, and increased levels of pro-allergic signatures [119,122]. It has been shown in mice there is a vertical transfer of microbiota where IL10^-/-^ gnotobiotic pregnant mice harboring an antibiotic-disrupted microbiota transfers their gut microbiota to their pups [123]. The pups having inherited a disrupted microbiota which has a higher incidence of colonic inflammation compared to those that inherited a controlled microbiota [123,124]. In humans, a recent study demonstrated that prenatal antibiotic treatment or neonatal infections disrupt not only the microbiota but also the normal T cell population developmental trajectory. In infants whose microbial colonization was disrupted, the risk for respiratory morbidity is significantly increased [76]. The immune development and the absence or presence of disease is dependent on microbial colonization and on the perturbations of the maternal microbiota. It is thus essential to fully understand neonate colonization and immune development.

**Figure 4:**
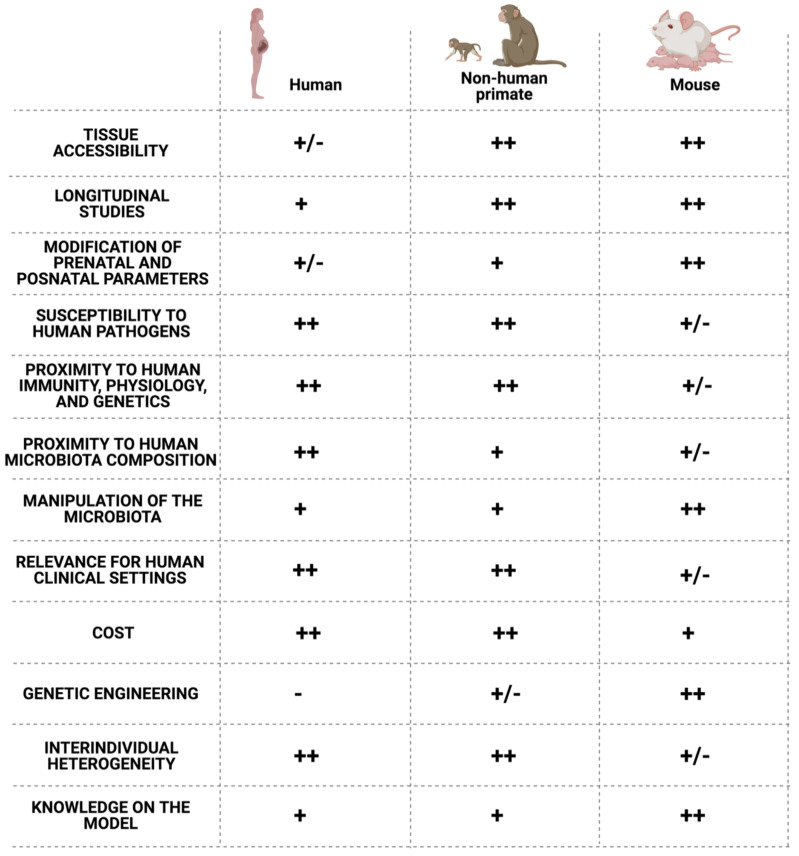
Comparison of the non-human primate and mouse models to the human. Created by BioRender.com, accessed on 20 April 2021.

Although it is still impossible to generate GF NHP models, the primates can help us answer complex questions that cannot be assessed in other animal models or in human studies [6] (Figure 3). Such questions may include the impact of antibiotic use in neonate colonization and immune system maturation and their link to infectious or non-infectious diseases in early life and later life [125]. The NHP model allows the monitoring of the microbiota’s early colonization in neonate, concomitantly with the bacterial metabolites produced and their respective role in shaping the immune system [126]. Indeed, as illustrated in Figure 4 below, the NHP model harbors several advantages for biomedical research.

What we know about the ontogeny of colonization is that the gut of a neonate is an aerobic environment that become anaerobic over time. The earliest colonizers include facultative aerobic bacteria such as *Staphylococcus*, *Streptococcus*, *Lactobacillus*, *Lactococcus*, and Enterobacteria [79,127] (Figure 1b). Oxygen consumption enables a shift to an anaerobic microbiota, including *Clostridiaceae*, Bacteroidetes, and *Bifidobacteria.* These bacteria are also found in aseptically collected breast milk [128]. The first 100 days of life seem to be a critical time-lapse for the establishment of relationships between the host and its microbiota [129]. Gut dysbiosis in neonates was associated with disease development [130,131]. Infants experiencing gut bacterial dysbiosis in the first two months of life had more circulating endothelial cells and activated T cell populations [73]. Newborn immune cell populations are very variable during the first three months of life where Olin et al. observed a transient expansion of monocytes, a gradual reduction in neutrophils, and an increase in CD4+ and CD8+ T cells upon birth followed by an increase in B cell abundance from one month of life [73]. In addition, neonate transcriptomic analysis revealed that between one and three months of life, the major upregulated genes are major histocompatibility complex (MHC) class II genes that are induced by IFN-γ upon microbial stimulation [73]. These results suggest that the first interactions with microorganisms are important for the development of the immune system very early in life. These interactions seem to allow the maturation of several innate immune cells (DCs and Natural-Killer NK cells) and naive B cells that acquire a phenotype close to the adult phenotype within the six months post birth (Figure 1C). In contrast, naive T cell phenotype varies a lot but does not converge to an adult phenotype during this period [73]. During the first six months of life, it is hard to clearly define a healthy baby microbiota. In fact, at an early age, babies’ gastrointestinal microbiota composition is unstable and variable over time. A more stable colonization begins together with food intake and then evolves according to the different phases of the diet (lactation and weaning) [132]. However, the more the baby ages, the more its microbiota resembles that of an adult. Palmer et al. showed that, indeed, one year old babies have very similar microbiota compared to their adult parents intestinal microbiota [79].

Breast milk, by its composition, plays a crucial role in the development of the immune system [133]. It contains epithelial and immune cells, soluble factors such as immunoglobulins (IgAs, IgMs, and IgGs), antimicrobial factors, cytokines, growth hormones, digestive enzymes in addition to viable bacteria, and bacterial DNA [128,134,135,136,137]. Human milk also contains lactoferrin and, in addition to its function of iron binding and transport, has an important role in neonates where it has broad-spectrum antimicrobial activity and prevents invasive fungal infections [138]. The qualitative and quantitative composition of breast milk changes among women but also during the lactation period (between colostrum, transition, and mature milk for example) [89]. The composition and evolution of the milk is similar in various primate species and partly depends on the relative amount of fat and protein in the milk [139]. It is also different for women who give birth at term or preterm [140]. A comparison of several studies on bacteria composition indicates that *Staphylococcus*, *Streptococcus*, *Pseudomonas*, *Bifidobacterium*, *Propionibacterium*, *Bacteroides*, *Corynebacterium*, and *Enterococcus* are the most cited dominant genus in human milk [141]. In addition, there is evidence of a vertical transfer of bacteria between mother to infant via breastfeeding, where up to 88% of genera are shared between breast milk and infant fecal samples [90,142].

Breast-fed babies are exposed to multiple maternal components that shape their colonization and early immune response and maturation. It has been clearly shown in mice models that the transfer of maternal immune cells via the colostrum at the beginning of lactation not only gives the infant protective immunity through supplementation of Ig production but also modulates the infant’s immune responses such as the modulation of the T cell mediated response and the development of the T cell repertoire [143]. As an example, IgA contained in breast milk contributes, together with neonatal Treg cells, to the suppression of T helper lymphocyte maturation at the level of the intestinal mucosa [144]. This process is maintained until weaning and allows the promotion of a pro-tolerogenic environment at the level of the infant intestinal mucosa [111] in favor of host/microbiota interactions. In addition, many cytokines such as IL-2, IL-6, IL-10, Tumor Growth Factor β (TGF-β), and Epidermal Growth Factor (EGF) present in breast milk have an immunomodulatory capacity and may influence the development of the neonatal immune system [133,140]. For example, IL-10 is a known anti-inflammatory cytokine that inhibits blood lymphocyte proliferation [145]. TGF- β regulates T cells, B cells, NK cells, macrophages, and DC functions [146]. However, few studies have presently investigated the influence of the cytokines in a relevant context of neonatal immune system maturation. Most of the studies were carried out on cord blood which appears to be a bad predictor for postnatal immune cell maturation [73]. The microbiota in the breast milk also provides a significant number of microbial antigens capable of stimulating the infant immune system. Breast milk also protects the intestinal mucosa of the newborn, which has low resistance to bacterial colonization, thus preventing the risk of colonization by pathogens. For example, it has been demonstrated that the expansion of *Enterobacteriaceae* is restricted by the IgA contained in breast milk, thus avoiding inappropriate immune stimulation that leads to a lower risk for Necrotizing Enterocolitis (NEC), which is a disease particularly common in non-breastfed premature children [147].

In comparison with formula fed babies, breastfed babies are colonized mainly by *Bifidobacterium* species (*B. breve* and *B. bifidum*) and *Lactobacillus* [148,149]. Breastfed babies exhibit a lower α-diversity at 40 days of age, with a low abundance of *Veillonella* and *Clostridioides*. However, when the bacteria diversity rises at six months of age, the breastfeeding associated differences are no longer observed [148]. In the NHP, studies showed no differences in the diversity of the gut microbiota between breastfed and formula fed infants before three months of age but the relative abundance of various bacteria genus such as *Clostridioides* and *Prevotella* was significantly different between the two groups [149]. This was associated with a different immune system development, with breast-fed infants developing more memory T cells and T helper 17 (Th17) cells than bottle-fed infants [150]. Differences between the groups persisted throughout the first year and could even be found in the fifth year of life [151]. These studies show that infant feeding practices can impact immunity years after birth.

## 5. Weaning and Food Diversification

Weaning is defined as the transition period of gradually introducing solid diet to an infant while withdrawing the supply of breast milk. It has been demonstrated that the microbial diversity in the infant’s gut increases drastically during weaning [152,153,154] concomitant with an important immune reaction at the level of the intestinal mucosa. There is also an association of the cessation of breastfeeding; the introduction of food; the decrease in the abundance of *Lactobacillaceae*, *Bifidobacteriaceae*, *Enterococcaceae*, and *Enterobacteriaceae*; and the increase in abundance and in *Lacnospiraceae* (*Blautia*, *Roseburia*, *Pseudobutyrivibrio*, *Dorea*, *Coprococcus*, and *Lachnospiraceae incertae sedis*), *Ruminococcaceae* (*Faecalibacterium* and *Ruminococcus*), and *Clostridiaceae* [153,155].

In mice, the decrease in EGF in breast milk at the end of the lactation phase (between the tenth and twentieth day after birth in mice) leads to an opening of GAP junctions, an increased intestinal permeability, and promotes the exposure of microbial antigens to antigen presenting cells of the intestinal mucosa [110]. Bacterial antigens are taken up in the mucosa by dendritic cells of the lamina propria. Under non-inflammatory conditions, CD103 dendritic cells can induce T cell activation and promote the development of a tolerogenic response [145]. Food and microbial antigens (intestinal microbiota and microbial metabolites) are of great importance during this period as they are necessary for the proliferation of Retinoic-acid-receptor-related orphan nuclear receptor gamma (ROR-γt) Treg cells mediated by Myeloid Differentiation Factor 88 (MyD88). This reaction is called the weaning reaction. It induces tolerance to the various antigens encountered during this period and the immune system is “imprinted” by the microbiota and by food antigens [156]. This weaning reaction is limited in terms of time: in mice, the reaction involves Signal Transducer and Activator of Transcription 3 (STAT3) activation, which is induced only transiently by microbial antigens at weaning. CD4+ T cell reacts to the expanding commensal microorganisms and STAT3 phosphorylation is rapidly extinguished [157].

Interestingly after weaning the infant microbiota is not fully stable and significant changes in bacterial abundance occurs [155]. Several genus such as *Prevotella*, *Acinetobacter*, *Desulfovibrio*, *Veillonella,* and *Clostridioides* tended to appear and disappear during the infant’s first year of life [79] but as long as the breastfeeding continues, at least partially, there is a presence of *Bifidobacterium* and *Lactobacillus* [77,153]. The anaerobic bacteria present towards 12 months of age are efficient fiber and carbohydrate degraders, which indicates an adaptation towards an adult-like microbiota. It is not until three years old that changes in the bacterial abundance stabilize [155]. The variations in the composition of the microbiota during weaning also determine the modalities of exposure to microbial antigens and thus the outcome in terms of immune maturation. As an example, the intestinal microbiota in Russian infants is less rich in *Bacteroides* spp. compared to Finnish and Estonian infants. They exhibit a reduced risk for autoimmune diseases that is more common in Finnish and Estonian infants. In fact, in Finnish and Estonian infants, exposure to LPS arises primarily from *Bacteroides* which is a potent immune activator [158].

In addition, the timing of weaning is critical. In fact, a delayed exposure to diversified food (and thus a prolonged breastfeeding) has long-term health consequences with an increased susceptibility to immune pathology. A good example of the importance of the timing in the induction of tolerance by food diversification is shown in babies that were introduced to gluten-containing foods from four months of age (during lactation). These babies had, indeed, a reduced risk of coeliac disease later in life compared to when it was introduced after six months of age (after lactation stops) [159,160]. Late exposure to gluten does not allow gluten tolerance because the weaning reaction window is closed at six months [160]. Gluten therefore causes a chronic inflammation in a significant number of children that leads to coeliac disease later in life. This example shows that the weaning reaction consists in a second important window of opportunity for immune maturation. In fact, given that the infant microbiota is not stable until around three years old, during this period there might be a chance for microbial modulation and colonization. Studies in mice also emphasize the importance of this timing, since after the weaning reaction immune tolerance is less easy to induce [161]. The exact timing of this window is still under debate but in humans the window situates somewhere around the end of lactation (6 and 24 months old). Given that several immunological parameters such as tolerance are directly linked to this “window of colonization opportunity” period, it is of essential importance to clearly define the timing. This can be studied in the NHP model where the timing of food diversification and the cessation of lactation can be controlled [150,162]. In addition, the exact composition of the solid food components can be equally studied, which would allow scientists to directly associate a microbiota composition to a better health outcome in later life.

## 6. Microbiota and Response to Vaccination in Neonates

The maturation of immunity in children is also achieved through vaccination. The World Health Organization (WHO) states that more than 1 billion children were vaccinated over the last decade, preventing 2 to 3 million deaths every year [163]. Vaccine schedules may differ from country to country but vaccination in newborns remain a gold standard worldwide for vaccines such as diphtheria-tetanus-pertussis (DTP) or Bacillus Calmette–Guérin (BCG) vaccines (https://vaccine-schedule.ecdc.europa.eu/, accessed on 20 April 2021). Nevertheless, even in countries with similar vaccine schedule, immunization varies greatly among vaccinated children [164]. For example, seroconversion rates to oral rotavirus (RV) vaccine is relatively low in low-income countries compared to high-income countries [165]. Similarly, response to hepatitis B, pneumococcal, or type B *Hæmophilus influenzae* (Hib) vaccination can vary greatly between vaccinated infants [166]. It is thus important to understand the role of the different parameters involved in the modulation of vaccine efficacy [167]. The intestinal microbiota composition appears to be important to ensure good immunological responses to oral vaccines [168]. Several reviews surveyed the role of the microbiota in the modulation of vaccine efficacy [165,169]. There are few studies in humans and even less studies on infants [170,171,172]. In infants, these studies show a positive association between a high relative abundance of Actinobacteria (for oral and parenteral vaccines) or Firmicutes (for oral vaccines) and a high humoral and cellular vaccine response. On the contrary, Proteobacteria (oral and parenteral vaccines) and Bacteroidetes (oral vaccines) were associated with lower responses to vaccination. A more recent study showed that Actinobacteria is enriched in the gut microbiota of infants with good mucosal IgA responses to oral poliovirus vaccination. However, IgA-negative infants demonstrated a higher diversity and a higher abundance of Firmicutes (more particularly in the class of Clostridia) compared to IgA-positive infants [173].

The mechanisms of microbiota modulation of vaccine responses are poorly understood. Currently, the studies that examined this question are only addressed in adult subjects. An important study on this topic is the phase IIb clinical trial for a recombinant adenovirus-based HIV vaccine (HIV Vaccine Trials Network 505). The authors demonstrated that naive CD4 T and B cells were imprinted by intestinal microbiota-antigens, and that there was a cross-reactivity between intestinal microbiota antigens and vaccine antigens. This led to an intestinal microbiota/HIV-1 cross-reactive immune response that was not efficient in protecting a subject from HIV-1 infection [174]. The failure of this clinical trial raised awareness and underlined that the intestinal microbiota must be considered when choosing epitopes for adult vaccination since cross-reactivity can be an issue. This may also be the case for infant vaccines and further studies are needed to elucidate the interactions between the microbiota and the vaccine responses in infants.

The reverse effect of the vaccination on the microbiota has also to be taken into account. Vaccination could alter the microbiota composition but this effect seems to depend on the vaccine and on the microbiota. For oral rotavirus vaccination, the vaccine does not seem to have any impact on the human infant gut microbiota [175,176]. In a NHP model, two anti-influenza vaccine schedules had little impact on the gut microbiota of infant and juvenile rhesus macaques [177]. On the contrary, in the adult NHP, a mucosal SIV or HIV-1 vaccination protocol has been shown to alter the rectal microbiome. In vaccinated rhesus macaques, the rectal microbiome was strongly correlated to vaccine-induced immunity [178,179] and to viremia control [179]. These studies demonstrate the need to look at the microbiome in vaccine design and evaluation. More precise NGS studies and a resolution at a species or strain level would be useful to assess (and further manipulate) the infant’s immune response to vaccination and the impact of vaccination on the microbiota.

Early life macaque immunization and longitudinal follow-up of the microbiota and the immune response to vaccination in different compartments would greatly improve the understanding of how the microbiota and its perturbations modulate vaccine efficacy [180].

## 7. Discussion

In this review we have highlighted the link between the mother microbiota, the neonate colonization, and its impact on its immune system development. We have synthetized the effects of the mother’s vaginal and intestinal microbiota on the early neonate colonization potentially affecting its health and immune responses. Even if it is not clear whether if the intrauterine and fetal tissues are actively colonized by bacteria, we must highlight that there is indeed an exposition to maternal and exogenous antigens including bacteria-derived molecules. It is thus important to maintain healthy microbiotas when pregnant to minimize the potential detrimental effects due to dysbiosis, both on the mother and on the baby. On one hand, pregnant women, have dramatic hormonal shifts that alter their gut microbiota, intestinal function, and transit [181]. Alterations of the future mother microbiota has been associated to excessive gestational weight gain, obesity, metabolic disorders, increased risk of gestational diabetes, fetal macrosomia, or preeclampsia [182]. Children born to mothers with obesity or diabetes have reduced abundance of *Lactobacillus* in their gut, which is a biomarker of negative health outcomes [183]. On the other hand, the vaginal microbiota of the mother has a higher stability during pregnancy [9,10,11]. In addition, there is an association between inflammation—and potentially vaginal dysbiosis—and the induction of premature labor [61,62,184]. It is thus essential to consider the use of prebiotics or probiotics during pregnancy that may have a positive effect in the mother’s health and, by consequence, on the babies’ outcome.

Several clinical studies where pregnant women utilize known probiotic species that include *L. paracasei*, *L. plantarum*, *L. rhamnosus* GG, *L. acidophilus*, *Streptococcus thermophilus*, and *Bidifobacterium* species have shown a prevention or a reduction in the incidence of bacterial vaginosis and of spontaneous preterm delivery; and to improve the glucose metabolism, blood pressure, and the incidence of preeclampsia [185,186,187]. However, some studies have shown no effect [188,189]. The difficulty for assessing and comparing the impact of probiotic use is mainly due to the difference between studies in women ethnicity, administration route, duration and type of treatment, bacteria quantity, and the moment of use which are all parameters that will impact colonization. These differences may explain the absence of clear results on probiotic use. In addition, the use of lactobacilli strains that are not directly issued from the vaginal cavity and are not any of the species identified as the dominant lactobacilli of the vaginal microbiota (e.g., *L. crispatus*, *L. gasseri*, and *L. jensenii* in particular) is a major point to be considered. The ideal vaginal probiotic would be a locally used product, preferably a stable strain of *L. crispatus*—the species associated with the best vaginal health outcome [190]—in sufficient doses (10^8^ minimum), to be administrated vaginally and to be used at least one full week off of the period when dysbiosis is present.

Considering the use of probiotics on pregnant women for beneficial effects on the fetus and newborn, clinical studies mainly point out beneficial effects on prenatal diseases as previously mentioned (preeclampsia, obesity, glucose metabolism, and preterm delivery) but not on the longitudinal outcomes of the baby. What we do know is that there is an exposition of the fetus to maternal and bacterial molecules [3]. During intrauterine gastrointestinal tract development, the fetus swallows increasing amounts of amniotic fluid that range from 200–250 mL/kg/day on the third trimester [191,192] and the ingestion contributes to the gastrointestinal tract development. Given that there is evidence of cell free DNA presence in the amniotic fluid, there is no evidence suggesting that this DNA originates from viable bacteria and thus it is difficult to believe that mother ingestion of probiotics would directly impact neonate gut maturation.

Considering the high intestinal permeability in the fetus [193], it would be interesting to assess the potential use of the amniotic fluid and uterine space as a therapeutic target. Further studies need to be conducted on the pharmacokinetics of potential antigens or bacterial metabolites crossing the materno–fetal membranes that could have a positive impact on the health of the fetus.

During the late stages of pregnancy and after birth, the mother’s gut is more permeable. The hypothesis arose of an active migration or translocation of bacteria to the mammary glands vectored by immune cell migration from one site to another [194,195]. In addition, bacteria found in breast milk have shown immunomodulatory properties that could contribute to the proper development of the neonate immune system [196]. For example, in a NHP model, *Bifidobacterium animalis* supplementation during formula feeding has shown to impact the gut microbiota, the metabolome, and the immunity of infants [197]. If these findings are confirmed and considering that during the neonatal period the microbiota is particularly unstable, it might be appropriate to modify the mother microbiota to reach the newborn through lactation. In any case, it is necessary to study the mechanisms involved in the modulation of the fetal immune system by potential probiotic candidates.

Animal models will be required in order to have access to materno–fetal interfaces during pregnancy and to study mucosal immunity maturation in newborns. The NHP has been successfully used to study and manipulate this interface [198] and represents a relevant animal model that provides privileged access to a consequent amount of biological tissues.

When it comes to infants, the gut microbiota is more malleable during the first two years after birth. The use of probiotics can be used to correct alterations in the composition of the microbiota in newborns (for example, after antibiotic treatment) and to promote health outcomes [199]. Probiotic treatment could help enhance the immune response to many vaccines. However, up to the present point, probiotic bacteria seems to have a moderated effect on immune responses to vaccines since few clinical trials showed a significant effect of probiotic treatment on vaccine response [200,201], even if probiotics do have an impact on intestinal immune responses [168,202]. On the other hand, several studies have shown a positive impact of the use of probiotics on the neonate digestive system, immune system, and even in preventing necrotizing enterocolitis, which is an inflammation leading to high mortality and severe morbidity [203]. Probiotics can thus be exploited to improve the infants’ health.

We have to keep in mind that with probiotics, effects are not only species-dependent but also strain-dependent. Thus, the rising knowledge on strain-dependent beneficial effects in health and the insights on different diets enlighten the path for targeted microbiota manipulation for long-term health benefits.
